# A Randomized Controlled Trial of Balloon Dilation as a Treatment for Persistent Eustachian Tube Dysfunction With 1-Year Follow-Up

**DOI:** 10.1097/MAO.0000000000001853

**Published:** 2018-07-23

**Authors:** Ted A. Meyer, Ellen M. O’Malley, Rodney J. Schlosser, Zachary M. Soler, Jason Cai, Mark J. Hoy, Patrick W. Slater, Jeffrey L. Cutler, Roger J. Simpson, Michael J. Clark, Habib G. Rizk, Theodore R. McRackan, Christopher F. D’Esposito, Shaun A. Nguyen

**Affiliations:** ∗ Department of Otolaryngology–Head and Neck Surgery, Medical University of South Carolina, Charleston, South Carolina; † Entellus Medical, Inc., Plymouth, Minnesota; ‡ North American Science Associates (NAMSA), Shanghai, China; § Austin Ear Clinic, Austin, Texas; || Colorado Sinus Institute, Denver, Colorado; ¶ Ear Nose Throat and Sinus Clinic, LLC, North Platte, Nebraska; # Donald Guthrie Foundation, Sayre, Pennsylvania

**Keywords:** Balloon dilation, 7-item Eustachian tube dysfunction questionnaire, Eustachian tube, Eustachian tube dysfunction, In-office procedures, Patient-reported outcome measures, Randomized control trial

## Abstract

**Objective:**

Compare Eustachian tube balloon dilation versus continued medical therapy (control) for treating persistent Eustachian tube dysfunction (ETD).

**Study Design:**

Prospective, multicenter, randomized controlled trial.

**Setting:**

Tertiary care academic center and private practice.

**Patients:**

Diagnosed with medically refractory persistent ETD.

**Interventions:**

1:1 Randomization to balloon dilation or control. After 6 weeks, control participants had the option to undergo balloon dilation if symptoms persisted.

**Main Outcome Measures:**

Primary efficacy endpoint was the comparison between treatment arms in the mean change from baseline in the 7-item Eustachian Tube Dysfunction Questionnaire (ETDQ-7) score. Primary safety endpoint was complication rate.

**Results:**

Sixty participants were randomized (31 balloon dilation, 29 control). Mean (SD) change in overall ETDQ-7 score at 6 weeks was −2.9 (1.4) for balloon dilation compared with −0.6 (1.0) for control: balloon dilation was superior to control (*p* < 0.0001). No complications were reported in either study arm. Among participants with abnormal baseline assessments, improvements in tympanogram type (*p* < 0.006) and tympanic membrane position (*p* < 0.001) were significantly better for balloon dilation than control. Technical success was 100% (91 successful dilations/91 attempts) and most procedures (72%) were completed in the office under local anesthesia. Improvements in the ETDQ-7 scores were maintained through 12 months after balloon dilation.

**Conclusions:**

Balloon dilation is a safe and effective treatment for persistent ETD. Based on improved ETDQ-7 scores, balloon dilation is superior to continued medical management for persistent ETD. Symptom improvement is durable through a minimum of 12 months. Procedures are well tolerated in the office setting under local anesthesia.

Patients with Eustachian tube dysfunction (ETD) experience symptoms of ear pain and pressure, fullness, crackling/popping sounds, muffled hearing, and pain or discomfort with barometric changes. Traditional treatments include oral or topically applied nasal steroids. Pressure equalization (PE) tubes are also used to treat symptoms of ETD but are considered a temporary solution that does not treat the underlying pathology. Negative effects of PE tubes include infection, hearing loss, otorrhea, need for dry ear precautions, plugging or extrusion and need for replacement, the potential for permanent tympanic membrane perforation, scarring, and cholesteatoma.

Previous cadaver (1–3) and clinical studies (4–12) have provided evidence of the safety and effectiveness of balloon dilation for the treatment of ETD.

The objective of this randomized trial was to compare outcomes of Eustachian tube balloon dilation with control for treating persistent ETD. Follow-up through 12 months is reported for all participants who underwent balloon dilation.

## MATERIALS AND METHODS

### Study Design and Population

This was a prospective, multicenter, randomized controlled trial comparing balloon dilation to continued medical therapy (control) in patients with persistent ETD. The study was approved by an Institutional Review Board for each participating site and all participants signed informed consent before undergoing study procedures. The study was registered on www.clinicaltrials.gov: NCT02391584.

Participants 18 years and older were eligible for enrollment if they were diagnosed with ETD for 12 months or longer with 3 or more ETD symptoms (ear pain, ear pressure, tinnitus, cracking or popping in ears, muffled hearing, feeling that ears are clogged) and were refractory to medical therapy. Failed medical therapy was defined as a minimum of either 4 weeks of daily intranasal steroid spray or one completed course of an oral steroid within 12 months before study enrollment. Participants were required to have an overall Eustachian Tube Dysfunction Questionnaire (ETDQ-7) score of 3 or higher, representing moderate to severe symptoms (13).

Exclusion criteria included a history of head or neck surgery within 3 months; patulous Eustachian tube; ears tubes in place or an unhealed perforation; temporomandibular joint disorders; Menière's disease; chronic rhinosinusitis, allergies, or reflux disease not controlled with medication; or anatomic conditions that would prevent transnasal access to the Eustachian tube. All participants were required to have a computed tomography scan of the temporal bones and participants with evidence of carotid artery dehiscence were not eligible for the study.

### Assessments

All participants underwent the following assessments at baseline and all follow-up visits (6 wk; 3, 6, and 12 mo): ETDQ-7, tympanometry, otoscopy, and Valsalva maneuver.

The ETDQ-7 is a validated, standardized, 7-item patient-reported questionnaire to assess symptom severity associated with ETD (13). The 7 questionnaire items cover the following ear symptoms: pressure, pain, feeling clogged, cold/sinusitis problems, crackling/popping, ringing, and muffled hearing. The questionnaire is not specific for laterality of the affected ear(s). Each item is assessed on a scale of 1 (no problem) to 7 (severe problem), and an overall score, which is the mean of the 7 item scores, is calculated. Scores in the range of 1 to 2 indicate no to mild symptoms, 3 to 5 indicate moderate symptoms, and 6 to 7 indicate severe symptoms.

The tympanic membrane position (normal or retracted) was assessed by otoscopy in all participants. Improvement at follow-up was defined as a change from retracted at baseline to normal at follow-up. Each participant was asked to perform a Valsalva maneuver and they noted whether they were able to “pop” or “clear” their ears, indicating a positive maneuver. Improvement was defined as a change from negative Valsalva at baseline to positive Valsalva at follow-up. Tympanometry was performed on all participants and the tympanogram type reported. At follow-up, improvement was defined per Eustachian tube as a change from type B at baseline to type A or C at follow-up, or a type C at baseline to a type A at follow-up.

Additionally, pure tone audiometry (PTA) and air-bone gap were tested at frequencies of 500, 1000, 2000, and 4000 kHz at baseline for all participants and at 6 months postprocedure for participants who underwent balloon dilation.

### Randomization

Participants meeting enrollment criteria were randomized 1:1 to either balloon dilation or control. Randomized intervention assignments for each site were generated by an independent statistician using variable block size distributions. Clinical sites, treating physicians, and sponsor were blinded to the randomization schema. There was no masking of interventions after randomization.

Participants randomized to balloon dilation underwent their procedure and were evaluated at 6 weeks postprocedure. Participants randomized to the control group continued their baseline medical therapy for 6 weeks after randomization. After the 6-week evaluation period, participants in the control arm who continued to experience moderate to severe symptoms (overall ETDQ-7 score ≥3) were given the option to crossover to balloon dilation and were followed according to the balloon dilation treatment arm. The 6-week post randomization evaluation values were used as balloon dilation baseline for the crossover participants. Postprocedure follow-up is reported for all participants who underwent balloon dilation (randomized or crossover) at 6 weeks and at 3, 6, and 12 months.

### Balloon Dilation Procedure

Participants who underwent balloon dilation were treated with the XprESS ENT Dilation System (Entellus Medical, Plymouth, MN). The distal end of the device has an atraumatic rounded ball tip designed to provide tactile feedback while accessing the Eustachian tube. The distal end is reshapable to allow treatment of a variety of patient anatomies. For this study, the tip was bent to an approximate 45 degree angle at the 2-cm mark for accessing the Eustachian tube. This bend provides a positive stop to ensure the treatment area is confined to the cartilaginous portion of the Eustachian tube. The balloon is available in diameters of 5 to 7 mm and lengths of 8 and 20 mm; size selection was based on physician preference. The balloon was inserted through the nose into the Eustachian tube orifice, inflated to 12 atmospheres, and held for 2 minutes before deflating and removal.

The site of service for the procedure (office, ambulatory surgical center, or operating room [OR]) and the choice of anesthesia (general or local) were at the discretion of the treating surgeon and participant preference. For participants undergoing balloon dilation under local anesthesia, procedure pain scores were reported immediately after the dilation procedure on a visual analog scale of 0 (no pain) to 10 (worst pain). No concomitant procedures were allowed during the study procedure.

### Primary Endpoints

The primary efficacy endpoint of the trial was the comparison between randomization arms for the mean change in overall ETDQ-7 scores from baseline to 6 weeks.

The primary safety endpoint was the rate of complications, defined as the percent of serious adverse events related to the device or procedure.

### Other Short-Term Outcomes

Additional short-term outcomes included technical success, procedural details, and differences between treatment arms for changes from baseline in middle ear function tests (tympanic membrane position, Valsalva maneuver, and tympanogram type).

Technical success was defined as the percent of successful dilations/dilation attempts where the balloon device is successfully delivered to the target location, inflated, deflated, and withdrawn from the treated Eustachian tube.

Improvements in abnormal tympanic membrane position (retracted), abnormal Valsalva (negative), and abnormal tympanogram type (type B or C) were compared between study arms.

### Long-Term Outcomes

Postprocedure changes from baseline through 12-month follow-up in the mean overall ETDQ-7 score and middle ear function tests are presented for all participants who underwent balloon dilation (randomized or crossover). Changes from baseline to 6 months postprocedure in audiometry (PTA and air-bone gap) are also presented.

### Sample Size and Statistical Analysis

The primary efficacy endpoint hypothesis was that symptom improvement after Eustachian tube balloon dilation is superior to continued medical therapy (control). A minimum of 34 participants (17 per arm) was determined necessary to test the study hypothesis, assuming 80% power with a one-sided alpha of 0.025 (Student's *t* test), mean changes in the overall ETDQ-7 score of −2.15 for the balloon dilation arm and –0.85 for the control arm, and standard deviation (SD) of 1.3 for both arms.

One-sided Student's *t* test was used to compare symptom improvement between study arms (primary endpoint) with value of *p* < 0.025 considered statistically significant. Two-sided Student's *t* tests and Wilcoxon rank-sum tests were used to compare other continuous measures; *χ*^2^ and Fisher's exact tests were used to compare categorical measures between study arms. Changes from baseline to follow-up within each study arm were compared using two-sided paired *t* tests and Wilcoxon signed-rank tests for continuous measures and McNemar's or Bowker's tests for categorical measures. For statistical tests other than the primary efficacy endpoint, values of *p* < 0.05 were deemed statistically significant.

Mixed model for repeated measures (MMRM) was used to evaluate significance of the mean change in the overall ETDQ-7 scores over time. An unstructured correlation matrix was adopted to account for the repeated measures within subjects. Dunnett's *t* test was used for the multiple comparisons with baseline. A value of *p* < 0.05 indicated statistical significance.

Statistical analysis was performed by an independent statistician (J.C.).

## RESULTS

From August 2015 to June 2016, 60 participants at five US investigational centers were randomized to balloon dilation (n = 31) or medical therapy (control, n = 29). Two participants randomized to balloon dilation and two participants randomized to control did not complete the 6-week evaluation. Additionally, one participant undergoing balloon dilation failed to complete the ETDQ-7 at the 6-week visit, resulting in 55 participants (28 balloon dilation, 27 control) evaluable for the primary efficacy endpoint analysis. There are no statistically significant differences between the participants in the randomized arms in terms of demographics or baseline characteristics (Table 1). Baseline symptoms as reported by the participants are shown in Table 2 by study arm.

**FIG. 1:**
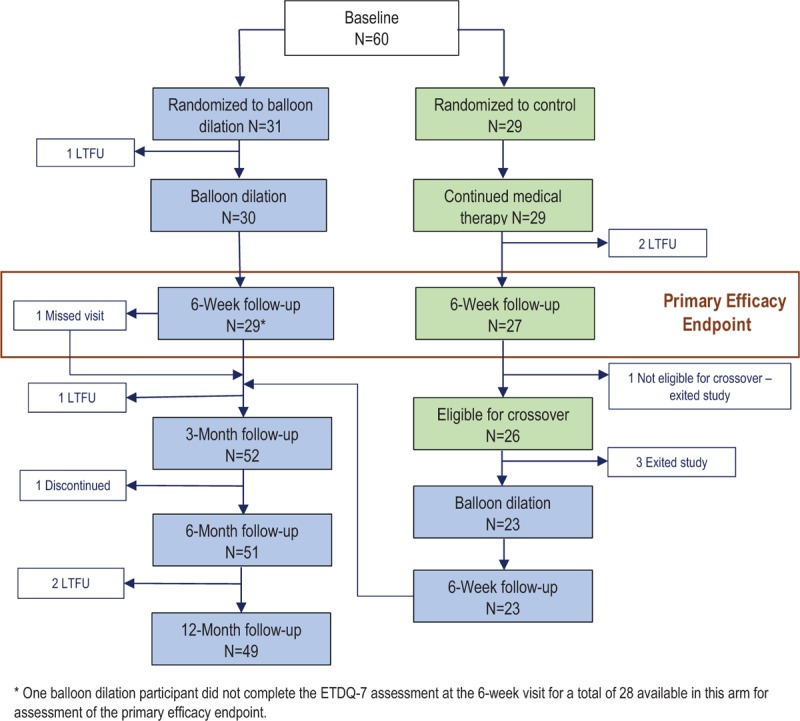
Participant flow diagram through 12-month follow-up.

Nearly all participants in both arms continued their baseline medications through the 6-week randomized period. Three participants in the balloon dilation arm discontinued nasal steroids within the 6-week period; all others continued their baseline medications. Of the 27 participants in the control arm who completed the 6-week evaluation, 26 (96.3%) were eligible for crossover to balloon dilation. One participant was ineligible for crossover to balloon dilation due to an improvement in ETD symptoms (ETDQ-7 score <3). Three of the 26 eligible participants chose not to undergo the crossover procedure, resulting in 23 participants in the crossover group. Figure 1 depicts the participant flow from randomization through the 12-month follow-up. Forty-nine of the 53 eligible participants completed the 12-month follow-up visit, and the overall follow-up visit compliance rate was 97% (284 actual/293 expected visits).

### Primary Endpoint Results

**FIG. 2:**
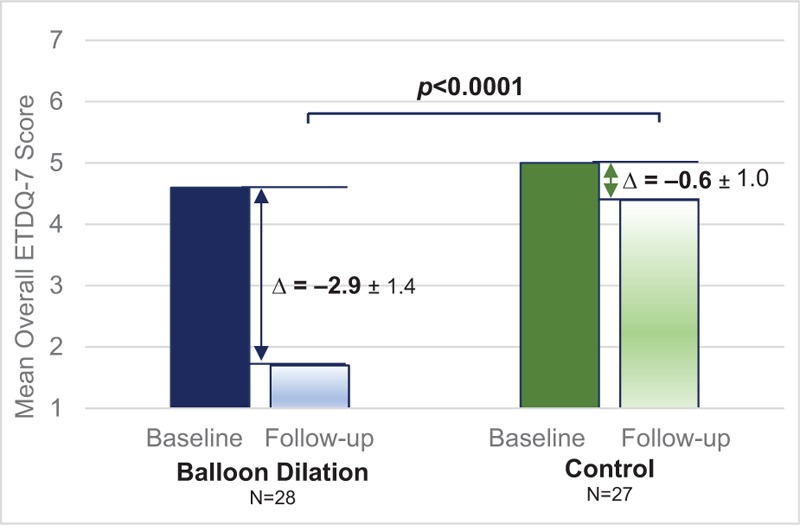
Primary efficacy endpoint: change in mean overall ETDQ-7 scores at 6 weeks by randomized treatment arm. *P* value is based on a one-sided, two-sample Student's *t* test comparing change from baseline between randomized arms with *p* < 0.025 indicating statistical significance. The change from baseline (Δ) is presented as the mean ± standard deviation. ETDQ-7 indicates 7-item Eustachian Tube Dysfunction Questionnaire.

The primary efficacy endpoint result for the mean change in the overall ETDQ-7 score between randomized arms at the 6-week time period is shown in Figure 2. The mean (SD) change in overall ETDQ-7 score is –2.9 (1.4) for balloon dilation compared with –0.6 (1.0) for control: the decrease in ETDQ-7 score is significantly greater for the group undergoing balloon dilation compared with the control group (*p* < 0.0001), demonstrating that balloon dilation is superior to the control.

The primary safety endpoint was the complication rate. No complications have been reported during the study.

### Procedural Information

Balloon dilation procedural data are shown in Table 3. A total of 91 Eustachian tube dilations were attempted in 53 participants (30 randomized to balloon dilation, 23 crossover to balloon dilation). All dilation attempts were successful, resulting in a technical success rate of 100% (91/91 Eustachian tubes). The majority of participants (38/53, 71.7%) were treated in the clinic setting under local anesthesia with a mean pain score of 4.1 on a scale of 0 to 10.

The specific local anesthesia protocols used were at the discretion of the surgeon. The typical local anesthesia protocol included preprocedure oral sedatives or anti-anxiolytics (e.g., hydrocodone/acetaminophen, diazepam) and decongestant/anesthesia sprays (e.g., lidocaine with neosynephrine). At the start of the procedure, anesthesia-soaked pledgets with or without 1:1,000 epinephrine were placed and a 1:1 solution of 1% lidocaine with 1:100,000 epinephrine was injected locally. The most frequent injection locations were the sphenopalatine, inferior turbinate, and middle turbinate. Allowing several minutes for the anesthetic to take effect before starting the procedure is critical for good pain management.

### Middle Ear Functional Assessments

Although the study enrollment criteria did not require participants to have abnormal results for tympanogram type, otoscopy (tympanic membrane position), and/or a negative Valsalva maneuver at baseline, these measures were collected and analyzed. The results for the randomized cohort are shown in Table 4.

For the participants with retracted tympanic membrane position at baseline, 66.7% (10/15) of those undergoing balloon dilation showed an improvement at 6 weeks (*p* = 0.002) versus 0% (0/12) of the participants in the control arm (*p* = not significant [NS]). The comparison between arms indicates that balloon dilation is significantly better than control for improvement in tympanic membrane position (*p* < 0.001). Of the participants in the balloon dilation arm who had a negative Valsalva maneuver at baseline, 47.1% (8/17) had significant improvement (positive Valsalva maneuvers) at 6 weeks (*p* = 0.005) versus 14.3% (2/14) of the participants in the control arm (*p* = NS). The difference between groups did not reach statistical significance (*p* = 0.068). Among the ears with type B or C tympanograms at baseline, 57.1% (8/14) of the ears treated with balloon dilation showed significant improvement at 6 weeks (*p* = 0.008) compared with 10.0% (1/10) of the ears in the control arm (*p* = NS). The difference between arms is statistically significant (*p* = 0.006) in favor of balloon dilation.

A post hoc analysis was performed to compare symptom improvement for participants with normal versus abnormal baseline middle ear functional assessments. The results demonstrate that improvement in ETDQ-7 scores was significantly better (*p* < 0.01 in all cases) for participants undergoing balloon dilation compared with participants in the control group, whether or not the participants had normal or abnormal tympanic membrane position, Valsalva maneuver, or tympanogram type at baseline.

### Long-Term Outcomes in All Participants Undergoing Balloon Dilation

**FIG. 3:**
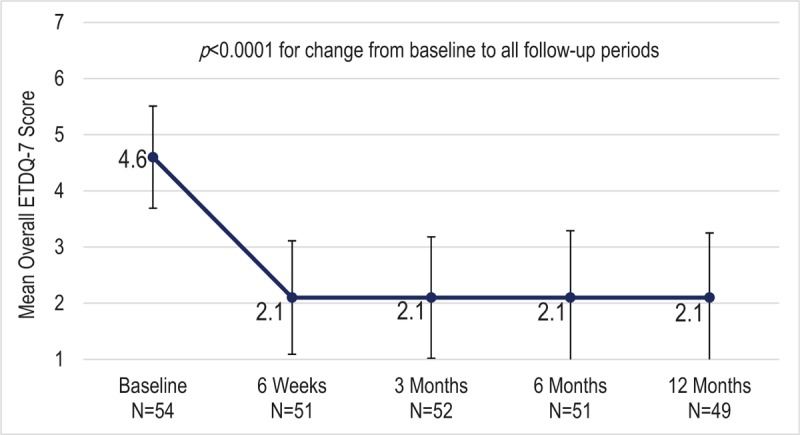
Mean overall ETDQ-7 scores over time for all participants undergoing Eustachian tube balloon dilation (randomized or crossover). *P* values are based on the mixed effects model of repeated measures (MMRM) analysis for the comparison between baseline and each follow-up visit. Error bars indicate standard deviations. ETDQ-7 scores of 1 to 2 indicate no to mild symptoms, 3 to 5 indicate moderate symptoms, and 6 to 7 indicate severe symptoms. ETDQ-7 indicates 7-item Eustachian Tube Dysfunction Questionnaire.

Figure 3 shows the mean overall ETDQ-7 score through 12-month follow-up among all participants who underwent balloon dilation (randomized and crossover). The mean overall ETDQ-7 score was significantly reduced from 4.6 at baseline to 2.1 (change = –2.5, *p* < 0.0001) at 6 weeks postprocedure and this reduction was maintained through the 12-month follow-up (*p* < 0.0001).

Table 5 presents the changes from baseline for the middle ear function assessments for follow-ups through 12 months postprocedure for all participants undergoing balloon dilation. The percentage of participants with normal tympanic membrane position improved from 51% to over 80% (*p* < 0.001 at all follow-ups). Ability to clear the ears with a Valsalva maneuver improved from 33% of participants to over 60% (*p* < 0.01 at all follow-ups). The percent of participants with type A tympanograms increased from 71 to 80% or more through 12-months with *p* < 0.05 at all but the 6-month time point (*p* = 0.139).

The participants with abnormal middle ear functional assessments at baseline experienced significant improvements in these assessments at 12 months. Normalization of the tympanic membrane position was experienced by 79.2% (19/24; *p* < 0.001), positive Valsalva maneuver by 62.5% (20/32; *p* < 0.0001), and improvement in tympanogram type by 55.0% (11/20; *p* = 0.006).

A post hoc subgroup analysis was performed on all participants who underwent balloon dilation. ETDQ-7 scores in participants with normal baseline middle ear function assessments were compared with those of participants with abnormal baseline assessments. The changes from baseline to 12-month follow-up are presented in Table 6 and demonstrate that both groups experienced clinically and statistically significant (*p* < 0.0001) improvements in all assessments. The differences between subgroups were not statistically significant for any of the middle ear functional assessments.

The shift in pure tone audiometry from baseline to 6 months was not statistically significant at any measured frequency. *P* values ranged from 0.451 to 0.995. The change in air-bone gap was not clinically significant at any frequency with all mean follow-up measurements within –2 to –0.4 dBa of the baseline for the frequencies measured.

During the study, two participants underwent additional ear surgeries for continuing or recurring symptoms. One participant (unilateral crossover) underwent a myringotomy with tube placement in the left ear due to recurring symptoms at approximately 3 months postprocedure. The procedure was performed at the same time as endoscopic sinus surgery for worsened chronic rhinosinusitis symptoms. This participant had a history of a previous tympanomastoidectomy of the left ear. The second participant reported undergoing a tympanostomy with paper patch placement for continued symptoms a few weeks before the 12-month study visit.

## DISCUSSION

We report the 12-month results from a randomized controlled trial of balloon dilation as a treatment for persistent ETD. Participants enrolled in the study were required to have been diagnosed with ETD for no less than 12 months, have 3 or more ETD symptoms, have an ETDQ-7 score 3 or higher, and have failed nasal steroid therapy for ETD. Furthermore, participants enrolled in the study were not allowed to have patulous Eustachian tube or uncontrolled CRS, allergies, or GERD. PE tubes and perforated tympanic membranes were also not allowed.

To evaluate severity of ETD symptoms, we used the validated ETDQ-7 survey (13). Although the ETDQ-7 is a subjective, patient-reported measure, we believe it is a very useful assessment of the severity of ETD. The tool has been shown to be responsive to improvements after treatment (7). In this study, statistically significant improvements in symptoms were seen after balloon dilation that demonstrated the superiority of balloon dilation over the control group. The baseline ETDQ-7 scores and postprocedure improvements over baseline that we observed are in agreement with other studies of patients with ETD (7,10). At baseline, the mean ETDQ-7 score of 4.6 for participants randomized to balloon dilation indicated symptoms were at the high end of moderate severity (3–5 on a 7-point scale). At 6 weeks after balloon dilation, the mean symptom score of 1.7 was improved (lower ETDQ-7 score) to within what is considered a normal level (≤2) (13). In contrast, the mean ETDQ-7 score of 5.0 for participants randomized to control was only reduced by –0.6 to 4.4 at 6-weeks, still within the moderate severity range. The authors caution that although the ETDQ-7 is beneficial for evaluating the change in severity of symptoms, it is not specific enough to be used alone to diagnose ETD. The decision for surgical intervention should be based on the entirety of the patient's clinical picture.

Enrollment criteria for this study did not require the participants to have abnormal results for otoscopy, Valsalva, and/or tympanogram at baseline, since ETD can be present even when these assessments are normal. In our study cohort, a retracted tympanic membrane was present in 45% of the randomized participants, a negative Valsalva was present in 55% of participants, while a type B or C tympanogram was present in 23% of the ears. Additionally, 33% of participants had previously had ear tubes placed. In total, 84% of the study participants who underwent balloon dilation had an abnormal finding for at least one of these three middle ear functional assessments or had previous ear tube placement. Among the participants/ears with abnormal baseline assessments, those in the balloon dilation arm demonstrated significant improvement and this improvement was significantly better than seen for participants in the control arm for tympanic membrane position and tympanogram type.

This study design included the option of a crossover from the control to balloon dilation after the 6-week evaluation period if symptoms were not improved. All but one of the 27 participants in the control arm were eligible for the crossover procedure as a result of continued ETD symptoms after 6 weeks of medical therapy. This further confirms that participants had persistent ETD and the inadequacy of continuing medical therapy for the treatment of persistent ETD. Procedure information for all participants undergoing balloon dilation, whether randomized or crossed over, is presented.

Previous studies providing evidence that Eustachian tube balloon dilation is safe and effective have been primarily studies of Eustachian tube dilation in the OR setting under general anesthesia (6–12). Luukkainen et al. (11) recently reported successful Eustachian tube balloon dilation in the OR setting using intravenous sedation instead of general anesthesia. Catalano et al. (6) reported use of topical anesthesia in a cohort of 26 patients undergoing Eustachian tube balloon dilation but only with dilation durations of up to 30 seconds. In our study, unlike the other Eustachian tube balloon dilation studies, the majority of the procedures were performed in the office setting under local anesthesia. Our study demonstrates that Eustachian tube balloon dilation under local anesthesia (topical and local injections) is well tolerated. All planned in-office procedures were completed in the office with dilation durations of 2 minutes per Eustachian tube and none of the procedures were stopped due to patient discomfort. The mean pain score is similar to that reported by Karanfilov et al. (14) for balloon sinus dilation and just slightly higher than that reported for other balloon sinus dilation studies (15).

Follow-up of all of the participants undergoing balloon dilation through 12-month follow-up period showed continued significant improvement in ETDQ-7 scores. In addition, the participants undergoing balloon dilation with abnormal middle ear assessments at baseline demonstrated significant improvement at the 12-month follow-up for tympanic membrane position, ability to clear the ears with a Valsalva maneuver, and tympanogram type. The improvements observed through 12-month follow-up in patient-reported symptoms and in objective middle ear functional tests confirm the efficacy of balloon dilation and demonstrate durability of the procedure.

Although carotid artery damage and patulous Eustachian tube are noted in the literature as potential adverse events associated with balloon dilation, these events have not been reported in the literature. The few adverse events that have been reported in the literature have been minor and transient in nature, e.g., bleeding, preauricular emphysema (6–12). This study provides additional evidence that Eustachian tube balloon dilation is a safe procedure for patients with ETD.

A limitation of this study was the inability to blind the participants to their treatment. This can lead to the placebo effect, especially with patient-reported outcomes. However, since we also observed significant improvements in objective findings such as tympanometry, otoscopy, and Valsalva maneuver in the balloon dilation arm and not in the control arm, we believe that any placebo effect is minimal and that the improvements observed in the ETDQ-7 scores are reliable and indicate true symptom improvement. The physicians were also not blinded to the participant's treatment assignment.

## CONCLUSION

Balloon dilation leads to a significant reduction in the mean overall ETDQ-7 score compared with control for patients with persistent ETD. Procedures are well tolerated in the office setting under local anesthesia. Of those participants with abnormal baseline measures, improvements in tympanogram type and tympanic membrane position were significantly better after balloon dilation compared with control. Statistically significant improvements in ETD symptoms and middle ear functional assessments (tympanic membrane position, Valsalva maneuver, and tympanogram type) were demonstrated at 12 months after balloon dilation. Eustachian tube balloon dilation is a safe, effective, and durable treatment for persistent ETD.

## Figures and Tables

**TABLE 1:** Demographics and baseline characteristics by randomized arm

Characteristic	Balloon Dilation	Control	*P* Value^*a*^
Age (yrs)	52.0 ± 15.4	46.6 ± 15.7	0.177
Sex (male)	45.2% (14/31)	51.7% (15/29)	0.796
Race			
Caucasian	90.3% (28/31)	89.7% (26/29)	>0.999
Other	9.7% (3/31)	10.3% (3/29)	
Never smoked	61.3% (19/31)	55.2% (16/29)	0.794
Medical history (participant reported)			
Allergies, perennial	32.3% (10/31)	41.4% (12/29)	0.469
Allergies, seasonal	32.3% (10/31)	37.9% (11/29)	
Previous ear tubes placed	45.2% (14/31)	20.7% (6/29)	0.058
GERD/LPR	25.8% (8/31)	17.2% (5/29)	0.536
Asthma	19.4% (6/31)	10.3% (3/29)	0.474
Diabetes	19.4% (6/31)	3.4% (1/29)	0.104
Chronic rhinosinusitis	9.7% (3/31)	13.8% (4/29)	0.702
Cholesteatoma	3.2% (1/31)	3.4% (1/29)	>0.999
Duration of ETD (yrs)	12.2 ± 17.0	13.0 ± 17.3	0.538
Baseline overall ETDQ-7 score	4.6 ± 1.1	5.0 ± 0.8	0.122
ETDQ-7 ≥3.0 at baseline	100% (31/31)	100% (29/29)	>0.999
Retracted tympanic membrane at baseline	48.4% (15/31)	41.4% (12/29)	0.614
Negative Valsalva maneuver at baseline	56.7% (17/30)+	53.6% (15/28)+	>0.999
Type B or C tympanogram at baseline (unit = ear)	26.9% (14/52)	19.2% (10/52)	0.486
Negative baseline middle ear function (retracted tympanic membrane, negative Valsalva, or Type B or C tympanogram) or previous ear tube placement	83.9% (26/31)	72.4% (21/29)	0.354

Numbers are mean ± SD for continuous measures and % (n/*N*) for categorical measures. *N* is participant unless otherwise specified. ET indicates Eustachian tube; ETD, Eustachian tube dysfunction; ETDQ-7, Eustachian tube dysfunction questionnaire; GERD/LPR, gastroesophageal reflux disease/laryngopharyngeal reflux.

^*a*^*P* values are based on Fisher's exact test for categorical measures and unpaired *t* test or Wilcoxon rank-sum test for continuous measures comparing the randomized balloon dilation arm with the randomized control arm.

**TABLE 2:** Baseline symptoms by randomized arm

Baseline Symptom	Balloon Dilation	Control	*P* Value^*a*^
Feeling of fullness	90.3% (28/31)	96.6% (28/29)	0.613
Ear pressure	90.3% (28/31)	93.1% (27/29)	>0.999
Muffled hearing	80.6% (25/31)	89.7% (26/29)	0.474
Popping noises	80.6% (25/31)	89.7% (26/29)	0.474
Crackling noises	80.6% (25/31)	86.2% (25/29)	0.732
Clicking noises	77.4% (24/31)	86.2% (25/29)	0.509
Head fullness	67.7% (21/31)	79.3% (23/29)	0.387
Tinnitus	67.7% (21/31)	75.9% (22/29)	0.573
Nasal congestion	74.2% (23/31)	65.5% (19/29)	0.576
Ear pain	58.1% (18/31)	82.8% (24/29)	0.050
Headaches	61.3% (19/31)	55.2% (16/29)	0.794
Barotitis	48.4% (15/31)	37.9% (11/29)	0.446
Dizziness	29.0% (9/31)	41.4% (12/29)	0.418
Increased voice resonance	25.8% (8/31)	44.8% (13/29)	0.176

Numbers are percents (count/*N*). *N* is participant.

^*a*^*P* values are based on Fisher's exact test comparing the randomized balloon arm with the randomized control arm.

**TABLE 3:** Procedure information for participants undergoing balloon dilation

	Randomized to Balloon Dilation	Control Crossed Over to Balloon Dilation	All Participants Undergoing Balloon Dilation
Procedure Details	*N* = 30^*a*^	*N* = 23	*N* = 53
Procedure location			
Clinic office	70.0% (21/30)	73.9% (17/23)	71.7% (38/53)
Ambulatory surgical center	26.7% (8/30)	17.4% (4/23)	22.6% (12/53)
Hospital outpatient	3.3% (1/30)	8.7% (2/23)	5.7% (3/53)
Anesthesia type			
Local anesthesia only	70.0% (21/30)	73.9% (17/23)	71.7% (38/53)
General anesthesia	30.0% (9/30)	26.1% (6/23)	28.3% (15/53)
Converted from local to general	0.0% (0/30)	0.0% (0/23)	0.0% (0/53)
Eustachian tubes treated			
Bilateral	66.7% (20/30)	78.3% (18/23)	71.7% (38/53)
Unilateral	33.3% (10/30)	21.7% (5/23)	28.3% (15/53)
Total Eustachian tubes treated	50	41	91
Technical success rate (unit = ET)	100% (50/50)	100% (41/41)	100% (91/91)
Procedure pain score^*b*^	3.4 ± 2.9 (21)	5.0 ± 2.5 (17)	4.1 ± 2.8 (38)
Total number of inflations (unit = ET)	1.0 ± 0.2 (50)	1.0 ± 0.0 (41)	1.0 ± 0.1 (91)
Total inflation time (min) (unit = ET)	2.1 ± 0.2 (50)	2.0 ± 0.0 (41)	2.0 ± 0.2 (91)

^*a*^One participant randomized to balloon dilation was lost to follow-up before undergoing the balloon dilation procedure.

^*b*^Pain assessments were evaluated for participants undergoing local anesthesia only. Score of 0 = no pain, 10 = worst pain. Numbers are mean ± SD (n) or percents (count/*N*). *N* is participant unless otherwise specified. ET indicates Eustachian tube.

**TABLE 4:** Change in middle ear function assessments from baseline to 6-week in randomized participants with abnormal baseline assessments

Status	Balloon Dilation	Control	Between Arm *P* Value
Tympanic membrane position			
Improved	66.7% (10/15)	0.0% (0/12)	<0.001^*a*^
Not improved	33.3% (5/15)	100% (12/12)	
Within arm *p* value^*b*^	0.002	–	
Valsalva maneuver			
Improved	47.1% (8/17)	14.3% (2/14)	0.068^*a*^
Not improved	52.9% (9/17)	85.7% (12/14)	
Within arm *p* value^*b*^	0.005	0.157	
Tympanogram type			
Improved	57.1% (8/14)	10.0% (1/10)	0.006^*c*^
No change	42.9% (6/14)	60.0% (6/10)	
Worsened	0.0% (0/14)	30.0% (3/10)	
Within arm *p* value^*d*^	0.008	0.625	

Results are presented as % (n/*N*). Tympanic membrane position improvement is defined as a change from retracted at baseline to normal at follow-up; *N* is participant. Valsalva maneuver improvement is defined as a change from a negative to a positive result; *N* is participant. Tympanogram type improvement is defined as a change from type B at baseline to type A or C at follow-up, or a Type C at baseline to a type A at follow-up; *N* is Eustachian tube.

^*a*^*P* values are based on the two-sided, two-sample Fisher's exact test for the difference between randomized arms at 6-week follow-up.

^*b*^*P* values are based on the two-sided, paired sample McNemar's test for the difference within each randomized arm.

^*c*^*P* value is based on the CMH *χ*^2^ test (row mean scores) for differences between randomized arms.

^*d*^*P* values are based on the Wilcoxon signed-rank test for differences within each randomized arm.

**TABLE 5:** Change in middle ear function assessments from baseline to 12-month postprocedure in all participants who underwent balloon dilation (randomized or crossover)

Middle Ear Assessment	Baseline	6 Weeks Postprocedure	3 Months	6 Months	12 Months
Tympanic membrane position					
Normal	51.0% (26/51)	84.6% (44/52)	84.3% (43/51)	82.4% (42/51)	85.7% (42/49)
Retracted	49.0% (25/51)	15.4% (8/52)	15.7% (8/51)	17.6% (9/51)	14.3% (7/49)
*P* value^*a*^	–	<0.001	<0.001	<0.001	<0.001
Valsalva maneuver					
Positive	32.7% (16/49)	61.2% (30/49)	63.3% (31/49)	69.4% (34/49)	66.0% (31/47)
Negative	67.3% (33/49)	38.8% (19/49)	36.7% (18/49)	30.6% (15/49)	34.0% (16/47)
*P* value^*a*^	–	0.004	0.001	<0.0001	0.001
Tympanogram type (unit = ear)					
A	71.3% (62/87)	89.7% (78/87)	83.1% (69/83)	80.0% (68/85)	87.5% (70/80)
B	11.5% (10/87)	3.4% (3/87)	7.2% (6/83)	8.2% (7/85)	5.0% (4/80)
C	17.2% (15/87)	6.9% (6/87)	9.6% (8/83)	11.8% (10/85)	7.5% (6/80)
*P* value^*b*^	–	<0.001	0.034	0.139	0.020

^*a*^*P* values are based on McNemar's test for the change from baseline for each time period.

^*b*^*P* values are based on Bowker's test for the change from baseline for each time period.

**TABLE 6:** ETDQ-7 score change from baseline to 12-months by baseline status of middle ear functional assessments in all balloon dilation participants

Middle Ear Assessment at Baseline	*N*	Baseline^*a*^	12 Months^*a*^	Change from Baseline^*a*^	*P* Value^*b*^	*P* Value^*c*^
Tympanic membrane position						
Normal	25	4.5 ± 0.9	2.1 ± 1.1	−2.4 ± 1.5	<0.0001	0.974
Retracted	24	4.5 ± 1.0	2.2 ± 1.2	−2.4 ± 1.3	<0.0001	
Valsalva maneuver						
Positive	15	4.4 ± 1.0	2.6 ± 1.1	−1.8 ± 1.2	<0.0001	0.052
Negative	32	4.5 ± 0.8	1.9 ± 1.1	−2.6 ± 1.3	<0.0001	
Tympanogram type						
Normal (type A)	32	4.7 ± 0.9	2.3 ± 1.2	−2.4 ± 1.5	<0.0001	0.972
Abnormal (type B or C)	17	4.3 ± 0.9	1.9 ± 1.1	−2.4 ± 1.2	<0.0001	

^*a*^Scores are reported as mean ± standard deviation.

^*b*^*P* values are based on paired *t* test for the comparison between baseline and 12-month follow-up.

^*c*^*P* values are based on unpaired *t* test for the comparison of the mean change in ETDQ-7 score between subgroups. Significance level is 0.05. ETDQ-7 indicates the 7-item Eustachian Tube Dysfunction Questionnaire.
